# Castration in Hypertrophy of the Prostate Gland

**Published:** 1895-02

**Authors:** 


For Texas Medical Journal.

### CASTRATION IN HYPERTROPHY OF THE PROSTATE GLAND.

[The University Medical Magazine for this month, February, '95, will say, editorially:

WHEN Dr. J. William White first suggested to the profession the operation of castration for the relief of hypertrophy of the prostate gland (Address at the annual meeting of the

American Surgical Association, June 1, 1893, *Annals of Surgery*, August, 1893) on theoretical grounds, although strongly supported by experimental evidence, it is doubtful whether any one appreciated the full value of the recommendation. Cases of prostatic hypertrophy are of extreme frequency. Sir Henry Thompson found that one man of every three over 54 years of age examined after death, showed some enlargement of the prostate; one in every seven had some degree of obstruction present; while one in fifteen had sufficient enlargement to demand some form of treatment. In this country to-day, as shown by the last census, there are more than three millions of men over fifty-four; of these, according to Thompson's estimate, which genito-urinary specialists consider a conservative one, about two hundred thousand are sufferers from hypertrophy of this gland. This number seems very large, but the assertions of Thompson unquestionably express a general rule, and in fact every surgeon must have seen men in whom some prostatic overgrowth existed *before* the fifty-fourth year. The lives of such patients are threatened because, if the obstruction is not removed, the health is rapidly undermined by the retention of urine, and the consequent fermentative changes, the deleterious influence of backward pressure on the kidneys, the frequent use of the catheter, and the loss of sleep incident to the incessant demands to void urine. Heretofore the surgeon has been unable to afford distinct relief from the distressing symptoms of an advanced case of this affection. If the patient's general condition would warrant the very considerable risk, some form of prostatectomy was performed. The suprapubic method was recommended for a time, but the difficulties encountered in its performance, the frequency of suprapubic fistula as a sequel, and the high mortality following the operation have led to its almost total abandonment. Perineal prostatectomy is also attended with considerable risk, on account of the free hemorrhage, which can not be controlled during the operation, and the prolonged anesthesia which is necessary. In addition to this, the operation is a bungling one, in which the enlarged gland is removed by cutting, scraping, or gouging, while the instrument is out of sight, and much of the time it can not be guided even by the finger. Combined suprapubic and perineal prostatectomy enables the operator to reach and enucleate the gland with greater freedom, but it is an operation of such gravity that it would be contraindicated in the very cases in which the demand for relief was most urgent.

Perineal prostatotomy is little more than a palliative measure, which does some good, temporarily, by draining the bladder and inducing slight contraction of the middle lobe of the prostate in the healing process. All of these operations confine the patient to bed for several weeks, which is, in itself, objectionable, and in addition require the use of the bougie for a long time afterwards.

In view of these facts, it is not strange that surgeons should have presented Dr. White's suggestion to patients suffering from the consequences of prostatic hypertrophy, nor is it unnatural that such patients accepted this chance for relief from a condition that in many cases was rapidly and surely impairing the health of a person otherwise vigorous and, apparently, without this trouble destined to enjoy many additional years of life.

With the testes already or soon to become functionless, and with the contemplation of a long period of intense suffering which will be relieved only by death, sentimental objections pale into insignificance, and the problem of securing relief without placing the life in danger is the only one entitled to consideration.

Cases of castration based upon Professor White's deductions soon began to be reported. Ramm, of Christiania, Norway, recorded two in September, 1893; Haynes, Los Angeles, Cal., and White, Philadelphia, each report three cases; Finley, Baltimore, reports two cases; Smith, St. Augustine, Fla., Powell, London, Mayer and Haenel, Dresen, Moullin, London, Thomas, Pittsburg, Ricketts, Cincinnati, Swain, Bristol, England, and Bereskin, Moscow, each record one case. Thus far eighteen operations have been published. All have been more or less successful, and usually the relief from the distressing symptoms and the shrinking of the prostate have been marvelous. The least favorable cases have experienced infinitely greater relief than has been obtained by any method heretofore employed. At least as many unpublished cases have been operated upon with equally favorable results. There have been no deaths from the operation: of course, few would be expected in the hands of competent surgeons.

To those familiar with these cases, the rapid shrinking of the prostate and the simultaneous relief afforded the patient have been truly wonderful. The operation has therefore passed the experimental stage, and has legitimately established for itself a position among the most successful operative procedures. Indeed, the results have been so uniformly favorable that castration may now be considered s specific for hypertrophy of the prostate.

It is necessary, however, to utter a word of caution here. Castration is not indicated in every case of prostatic enlargement or urinary obstruction. To secure uniformly successful results one must be certain that the condition from which the patient is suffering is appropriate for the operation. Cases of prostatic abscess, prostatitis, tumors of the prostate and of the region of the neck of the bladder, and other forms of obstruction in the neighborhood of the prostate must be distinguished from true prostatic hypertrophy. Without careful discrimination, both the surgeon and the patient will be disappointed, and the operation will unnecessarily be brought into discredit.

As it stands to-day, however, in appropriate cases, it appears to mark an advance in the surgery of the prostate, which, when the gravity and the frequency of the condition of hypertrophy are recalled, together with the more or less ineffectual and always dangerous methods of treatment which have prevailed, must be a source of congratulation not only to Professor White but to the profession at large, and to thousands of patients who, having outlived their sexual lives and earned an old age of mental and physical repose and intellectual enjoyment, have had only a few short years of torment and misery to look forward to on account of this hitherto intractable disease.

